# Standardization in the management of gram-negative bloodstream infections after implementation of a clinical care guideline at a large academic, safety-net institution: a quasi-experimental study

**DOI:** 10.1017/ash.2025.4

**Published:** 2025-02-12

**Authors:** Michael A. Deaney, Katherine C. Shihadeh, Alexandra Craig, Margaret M. Cooper, Paul D. Paratore, Timothy C. Jenkins

**Affiliations:** 1 Department of Pharmacy, Denver Health & Hospital Authority, Denver, CO, USA; 2 Department of Medicine - Infectious Disease, Denver Health & Hospital Authority, Denver, CO, USA

## Abstract

**Objective:**

To evaluate the impact of implementing a clinical care guideline for uncomplicated gram-negative bloodstream infections (GN-BSI) within a health system.

**Design:**

Retrospective, quasi-experimental study.

**Setting:**

A large academic safety-net institution.

**Participants:**

Adults (≥18 years) with GN-BSI, defined by at least one positive blood culture for specific gram-negative organisms. Patients with polymicrobial cultures or contaminants were excluded.

**Interventions:**

Implementation of a GN-BSI clinical care guideline based on a 2021 consensus statement, emphasizing 7-day antibiotic courses, use of highly bioavailable oral antibiotics, and minimizing repeat blood cultures.

**Results:**

The study included 147 patients pre-intervention and 169 post-intervention. Interrupted time series analysis showed a reduction in the median duration of therapy (–2.3 days, *P* = .0016), with a sustained decline (slope change –0.2103, *P* = .005) post-intervention. More patients received 7 days of therapy (12.9%–58%, *P* < .01), oral antibiotic transitions increased (57.8% vs 72.2%, *P* < .05), and guideline-concordant oral antibiotic selection was high. Repeat blood cultures decreased (50.3% vs 30.2%, *P* < .01) without an increase in recurrent bacteremia. No significant differences were observed in 90-day length of stay, rehospitalization, recurrence, or mortality.

**Conclusions:**

Guideline implementation was associated with shorter antibiotic therapy durations, increased use of guideline-concordant oral antibiotics, and fewer repeat blood cultures without compromising patient outcomes. These findings support the effectiveness of institutional guidelines in standardizing care, optimizing resource utilization, and promoting evidence-based practices in infectious disease management.

## Introduction

Gram-negative bloodstream infections (GN-BSI) pose a major healthcare challenge due to their high prevalence, morbidity, mortality and financial burden.^1–4^ Optimizing management through evidence-based practices is crucial to ensure effective treatment while minimizing adverse effects like antimicrobial resistance, *Clostridioides difficile* infections, catheter-related complications, and prolonged hospital stays.^5,6^ In 2021, Heil *et al* issued a consensus statement to standardize the management of uncomplicated GN-BSIs, defined as cases involving non-immunocompromised patients that achieved source control and clinical improvement within 72 hours.^7^ Common sources of uncomplicated GN-BSIs include the urinary tract, intra-abdominal, biliary tract, skin and soft tissue, pneumonia, or catheter-related infections. Key management points included 7-day treatment durations,^8–13^ transitioning to highly bioavailable oral antibiotics,^14–17^ and avoiding repeat blood cultures.^7,18–21^ The objective of this study was to evaluate the effects of implementing a clinical care guideline based on this consensus statement at a large academic safety-net institution.

## Methods

In September 2022, Denver Health Medical Center’s Antimicrobial Stewardship Program introduced a clinical care guideline for uncomplicated GN-BSI to standardize care and promote evidence-based management. Aligned with the consensus statement by Heil *et al*,^7^ the guideline emphasized 7-day treatment durations, limited use of repeat blood cultures, and transitioning from intravenous to highly bioavailable oral therapy after 24–48 hours of clinical stability. Recommended oral options included levofloxacin, trimethoprim-sulfamethoxazole (TMP-SMX), and cephalexin. Clinicians were advised to consider susceptibilities and clinical factors when selecting therapy, with high-dose cephalexin (1 gram given thrice daily) as the preferred oral beta-lactam due to its favorable pharmacokinetics and toxicity profile as well as narrow spectrum of activity.^7^ The guideline was accessible to clinical staff through the Denver Health Antimicrobial Stewardship Mobile Application and intranet. Awareness of the guideline was also promoted via a newsletter and direct communication from representatives of the Antimicrobial Subcommittee that approved the guideline. Infectious Diseases pharmacists reviewed positive blood cultures and provided feedback to clinicians to promote use of the guideline. Of note, an intervention to reduce routine use of repeat blood cultures via a best-practice advisory alert was implemented more than two years prior in January 2020.

This was a retrospective, quasi-experimental study comparing patients admitted with a GN-BSI before (January 2019–December 2019) and after (October 2022–September 2023) guideline implementation. The COVID-19 pandemic resulted in a delay in the development and implementation of the guideline and thus a gap between the two periods; however, this may have served to reduce potential confounding of outcomes during the early pandemic.^22–24^ Patients were included if they were over 18 years old, admitted during the study period, and had at least one positive blood culture with one of the following organisms: *Acinetobacter* spp., *Citrobacter* spp., *Enterobacter* spp., *Escherichia coli*, *Klebsiella* spp., *Morganella morganii*, *Proteus* spp., *Providencia stuartii*, *Pseudomonas* spp., *Serratia* spp., and *Stenotrophomonas maltophilia*. Patients with polymicrobial cultures or an organism thought to be a contaminant were excluded. Automated antimicrobial susceptibility testing was performed using the MicroScan system.

The primary outcome was the difference in antibiotic duration. Secondary outcomes included the proportion receiving seven days of therapy, oral switch therapy, and repeat blood cultures. Safety outcomes included length of hospitalization and 90-day incidence of rehospitalization, recurrence of GN-BSI (defined as a positive blood culture with the same organism as the index case), all-cause mortality, and *C. difficile* infection (defined as a positive polymerase chain reaction or toxin assay test). Patients who did not survive the initial hospitalization were not censored.

Trends in the primary outcome were analyzed using interrupted time series (ITS) analysis with an ARMA(0,1) regression model. R was used for development of the regression model and ITS analysis. The analysis was constructed using monthly median days of therapy with 12 points of data before and after the intervention. Other results were analyzed using the Mann-Whitney *U* test and χ^2^ test, with differences considered significant with a *P*-value of < .05.

This project was reviewed by the Denver Health’s Quality Improvement Committee, authorized by the Colorado Multiple Institutional Review Board (COMIRB), and deemed not human subject research, thus exempt from IRB review.

## Results

A total of 147 patients were included from the pre-intervention period and 169 from the post-intervention period. Baseline characteristics of patients and their infections are shown in Table 1. Age, gender, and Charlson comorbidity index scores were similar between groups. The median Pitt Bacteremia Score was one in both groups (*P* = .51), and there were fewer patients in the intensive care unit at the time of first blood culture in the post-intervention period (42.9% vs 32%, *P* < .05). *Escherichia coli* was the most common pathogen in both periods (over 50%), followed by *Klebsiella pneumoniae* and *Enterobacter cloacae*. More than half of infections originated from the urinary tract. There was no significant difference in the number of extended-spectrum beta-lactamase (ESBL)-producing organisms or resistance to oral antibiotics. No carbapenemase-producing organisms were detected. The frequency of Infectious Diseases consults was similar (about 30%) between groups.

**Table 1.** Comparison of baseline characteristics of included patients and their gram-negative bloodstream infections between cohorts

Characteristic		Pre-intervention n = 147	Post-intervention n = 169	*P*
Age (years), median (IQR)		62 (49–70)	58 (46–69)	0.35
Gender at birth, n (%)	Female	74 (50.3)	84 (49.7)	0.91
Race, n (%)	White	104 (70.7)	102 (60.4)	0.05
	Black	11 (7.5)	14 (8.3)	0.79
	Asian	8 (5.4)	5 (2.9)	0.27
	Other	24 (16.4)	48 (28.4)	<0.05
Ethnicity, n (%)	Hispanic	83 (56.5)	100 (59.2)	0.63
Charlson Comorbidity Index, median (IQR)		4 (2–5)	3 (1–5)	0.30
Comorbid conditions, n (%)	Injection drug use	8 (5.4)	8 (4.7)	0.77
	Solid organ transplant	4 (2.7)	2 (1.2)	0.32
	Bone marrow transplant	0 (0)	0 (0)	N/A
	Absolute neutrophil count <500 cells/microliter	1 (0.7)	1 (0.6)	0.92
Causative pathogens, n (%)	*Escherichia coli*	92 (62.6)	100 (59.1)	0.54
	*Klebsiella pneumoniae*	20 (13.6)	27 (16)	0.55
	*Enterobacter cloacae*	8 (5.4)	10 (5.9)	.86
	*Klebsiella oxytoca*	9 (6.1)	1 (0.6)	< 0.05
	*Pseudomonas aeruginosa*	7 (4.8)	5 (3)	0.40
	*Proteus mirabilis*	5 (3.4)	6 (3.6)	0.94
	Other^a^	6 (4.1)	20 (11.8)	< 0.05
Extended-spectrum beta-lactamase producing organism, n (%)		18 (12.2)	26 (15.4)	0.42
Antimicrobial rate of resistance, n (%)	Cefazolin^b^	65 (44.2)	64 (37.9)	0.25
	TMP-SMX	48 (32.7)	45 (26.6)	0.24
	Levofloxacin	23 (15.6)	21 (12.4)	0.41
Suspected source of infection, n (%)	Urinary tract	83 (56.5)	111 (65.7)	0.09
	Intra-abdominal	27 (18.4)	31 (18.3)	0.99
	Respiratory	10 (6.8)	10 (5.9)	0.75
	Vascular catheter	5 (3.4)	5 (2.9)	0.82
	Skin and soft tissue	3 (2)	6 (3.6)	0.42
	Other	19 (12.9)	6 (3.6)	< 0.05
Pitt Bacteremia Score, median (IQR)		1 (0–3)	1 (0–3)	0.51
Intensive care unit admission on day of positive blood culture, n (%)		63 (42.9)	54 (32)	< 0.05
Infectious Diseases consult, n (%)		48 (32.7)	51 (30.2)	0.64

IQR, interquartile ratio; n, number of members; %, percentage of sample size.

a *Acinetobacter baumannii*, *Citrobacter freundii, Citrobacter koseri, Enterobacter aerogenes, Morganella morganii, Salmonella enterica, Serratia marcescens*, and other unidentified species of *Enterobacter* and *Acinetobacter*.

b Susceptibilities for cephalexin and cefdinir are extrapolated from cefazolin per the 2024 Clinical and Laboratory Standards Institute M100 guidance.

**Figure 1.**
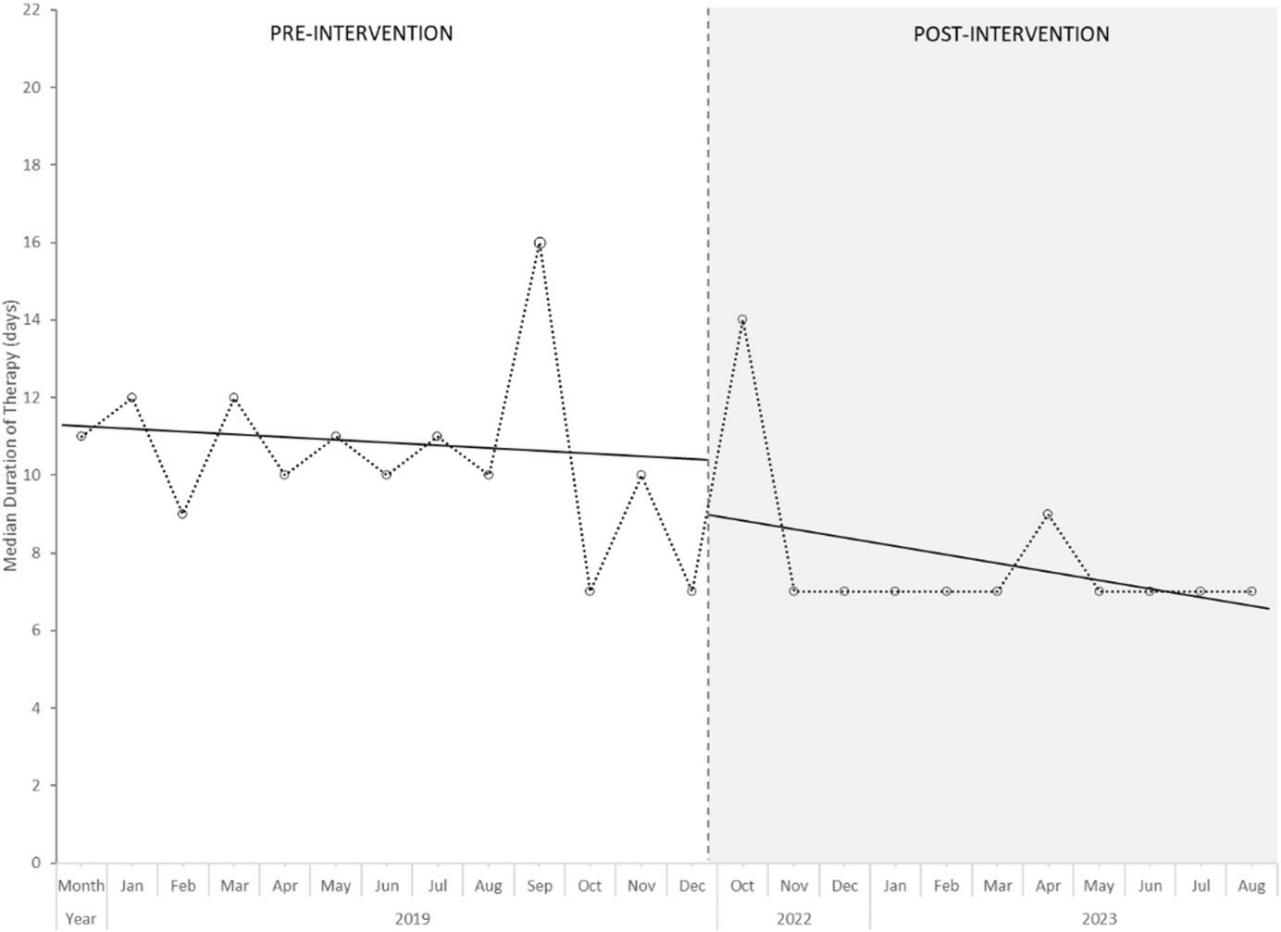
Interrupted time-series analysis of median duration of antibiotic therapy between pre-intervention and post-intervention periods.

The results of the ITS analysis are displayed in Figure 1. The initial median duration of therapy was 10.5 days and a significant level change (–2.3 days, *P* = .0016) was observed following the intervention. This was maintained after the intervention and the rate significantly decreased over the course of the post-intervention period (slope change –0.2103, *P* = .005). In aggregate, patients in the post-intervention period were more likely to be treated with seven days of antibiotics (12.9% vs. 58%, *P* < .01) and had a lower median duration of therapy (10 vs. 7 days, *P* < .01) (Table 2).

**Table 2.** Comparison of aggregate primary and secondary outcomes between cohorts

Outcome		Pre-intervention n = 147	Post-intervention n = 169	*P*
Total days of therapy, median (IQR)		10 (7.5 – 14)	7 (7 – 10)	< 0.01
Received seven days of therapy, n (%)		19 (12.9)	98 (58)	< 0.01
Initial use of intravenous therapy, n (%)		146 (99.3)	164 (97)	0.14
Use of oral switch therapy, n (%)	Any oral agent	85 (57.8)	122 (72.2)	<0.05
	Beta-lactam	42 (28.6)	45 (26.6)	0.70
	Fluoroquinolone	33 (22.4)	63 (37.3)	< 0.05
	Cefdinir	21 (14.3)	7 (4.1)	<0.01
	TMP-SMX	10 (6.8)	13 (7.7)	0.76
	Amoxicillin	8 (5.4)	2 (1.2)	< 0.05
	Cephalexin	6 (4.1)	26 (15.4)	< 0.01
	Amoxicillin-clavulanate	7 (4.8)	10 (5.9)	0.65
	Other oral agent^a^	0 (0)	1 (0.6)	N/A
Days to oral switch, mean (SD)		4.8 (2.4)	4.5 (3.7)	< 0.05
Use of repeat blood cultures, n (%)	Repeat blood cultures collected	74 (50.3)	51 (30.2)	< 0.01
	Positive repeat blood cultures for same gram-negative pathogen	3 (2)	4 (2.4)	0.84
	Cases with repeat blood cultures positive for a contaminant organism	7 (4.8)	3 (1.8)	0.13
Length of hospitalization, median (IQR)		5 (3–10)	4 (3–8)	0.15
90-day incidence of *C. difficile* infection, n (%)		7 (4.8)	1 (0.6)	< 0.05
90-day rehospitalization, n (%)		49 (33.3)	56 (33.1)	0.97
90-day recurrence of GN-BSI, n (%)		2 (1.4)	3 (1.8)	0.57
90-day mortality, n (%)		21 (14.3)	13 (7.7)	0.06

n, number of members; %, percentage of sample size; IQR, interquartile ratio; SD, standard deviation; GN-BSI, gram-negative bloodstream infection.

a Fosfomycin (n = 1).

The use of oral switch therapy was more frequent in the post-intervention period (57.8% vs 72.2%, *P* < .05), while the mean number of days to oral switch was lower (4.8 vs 4.5 days, *P* < .05). The use of oral fluoroquinolones (22.4%–37.2%, *P* < .05), and cephalexin (4.1%–15.4%, *P* < .01) were higher in the post-intervention period. Conversely, cefdinir and amoxicillin use was lower (14.3%–4.1%, *P* < .01 and 5.4%–1.2%, *P* < .05, respectively). The overall use of oral beta-lactams was not significantly different (28.6% vs 26.6%, *P* = .7). Repeat blood culture collection was lower in the post-intervention period (50.3% vs 30.2%), *P* < .01), with no significant differences in positivity rates for the same pathogen (2%–2.4%, *P* = .84). There was a trend toward fewer contaminated blood cultures in the post-intervention period (7 vs 3 cultures, *P* = .13).

No significant differences were observed in median length of stay or 90-day incidence of rehospitalization, recurrent GN-BSI, or mortality. However, 90-day incidence of *C. difficile* infection was significantly lower during the post-intervention period (4.8% vs 0.6%, *P* = .01).

## Discussion

Evidence-based local guidelines have been shown to improve the management of multiple infections, including but not limited to pneumonia, skin and soft tissue infections, and acute otitis media.^25–28^ In the present study, we demonstrate that implementation of an institutional clinical care guideline for uncomplicated GN-BSI was associated with an immediate and sustained reduction in the median duration of antibiotic therapy, highlighting the effectiveness of local guidelines as a tool to shorten treatment durations. We believe local guidelines increase clinician knowledge of evidence-based practices and serve to streamline and standardize decision-making. The addition of Infectious Diseases pharmacist review and feedback serves as an opportunity to promote use of the guideline and reinforce the management concepts with clinicians.

In addition to shorter durations of therapy, implementation of the guideline was associated with more frequent use of oral switch therapy. Furthermore, there was a notable shift toward guideline-recommended antibiotics, such as cephalexin and levofloxacin, and away from cefdinir. The shift from cefdinir to high-dose cephalexin was one intended goal of this intervention, as cefdinir’s poor bioavailability has been linked to worse outcomes in GN-BSI.^17^ The increased use of levofloxacin may have in part been due to the increasing incidence ESBL-producing organisms in our health system and other changes in resistance patterns associated with the COVID-19 pandemic.^29^ Future stewardship efforts could focus on promoting narrower-spectrum agents like beta-lactams when appropriate. These shifts in antibiotic selection reinforce the impact of local guidelines on prescribing practices.

The significant reduction in repeat blood cultures warrants further discussion. The introduction of a best-practice advisory alert discouraging routine repeat cultures prior to the intervention may have contributed to this change, though it is unclear whether the effect was due to the advisory, the guideline, or both. Importantly, there was no increase in recurrent bacteremia, indicating that reduced use of repeat cultures did not compromise patient safety. Additionally, the less frequent use of repeat blood cultures may have contributed to the lower number of contaminated blood cultures in the post-intervention period, a potentially important benefit since contaminated blood cultures have been shown to be associated with unnecessary antimicrobial use and prolongation of hospitalization.^30–34^ The absence of significant differences in length of stay, rehospitalization, recurrent infections, and mortality suggests that the guideline’s shorter therapy durations and reduced repeat blood cultures did not negatively impact patient outcomes.

This study has similarities to a prior study by Erickson *et al* who evaluated an antimicrobial stewardship bundle for uncomplicated GN-BSI prior to the publication of the consensus statement by Heil *et al.* ^7,35^ However, the present study differs by including a larger sample size, a diverse and underserved population, and a more recent study period, reflecting current prescribing practices and resistance trends. Both studies demonstrated reduced treatment duration and repeat blood culture use without increased readmissions, infection recurrence, or mortality, suggesting clinical care guidelines can be effectively and safely implemented across various settings.

Several limitations should be considered. First, the quasi-experimental study design limits the ability to establish a causal relationship between the intervention and practice changes. The time gap between the pre- and post-intervention periods introduces the potential for period effect (eg, other interventions or secular trends influencing outcomes). However, the consistent changes across multiple outcomes – such as duration of therapy, transitioning to oral antibiotics, oral antibiotic selection, and repeat blood cultures – suggest that the guideline was at least in part responsible for those shifts. It is unclear if the observed reduction in *C. difficile* infections was related to less antibiotic exposure because of this intervention or other organizational *C. difficile* reduction efforts, such as promoting testing only in patients with true diarrhea and avoiding testing patients who received laxatives, that were also ongoing during the post-intervention period. Second, due to retrospective design, we may have missed clinical outcomes such as deaths, readmissions, and recurrence of gram-negative bacteremia, though these would be expected to be similarly distributed across periods. Third, we did not control for factors that may influence clinical outcomes like appropriateness of initial therapy, location of infection onset (hospital vs community), and antimicrobial resistance. The limited number of ESBL- or carbapenemase-producing organisms may mitigate the latter concern. Finally, the study’s single center-design and limited sample of immunocompromised patients (ie solid organ transplant, neutropenia, etc) limit generalizability.

Implementing a clinical care guideline for uncomplicated GN-BSI significantly reduced antibiotic therapy duration without compromising clinical outcomes. It also promoted guideline-concordant oral antibiotic use and more selective use of repeat blood cultures. These findings underscore the potential of such guidelines to standardize care, enhance antibiotic stewardship, and optimize resource use in managing infectious diseases.

## Data Availability

Data not publicly available.
